# Qualitative Task Analysis to Enhance Sports Characterization: A Surfing Case Study

**DOI:** 10.2478/hukin-2014-0078

**Published:** 2014-10-10

**Authors:** Miguel Moreira, César Peixoto

**Affiliations:** 1 SpertLab, Sport and Health Department, Faculty of Human Kinetics, University of Lisbon.

**Keywords:** qualitative research, taxonomy, structural analysis, functional analysis, nature sports, sliding sports

## Abstract

The aim of this study was to develop a Matrix of Analysis for Sports Tasks (MAST), regardless of the sports activity, based on practice classification and task analysis. Being this a qualitative research our main question was: in assessing sports’ structure is it possible to make the characterization of any discipline through context and individuals’ behaviours? The sample was within a surf discipline in a competition flowing having 5 of the top 16 Portuguese surfers training together. Based on a qualitative method, studying the surf as the main activity was an interpretative study case. The MAST was applied in four phases: taxonomy; tasks and context description; task analysis; teaching and performance strategies. Its application allowed the activities’ characterization through the observation, surfer’s opinions and bibliographical support. The triangulation of the data was used as an information data treatment. The elements were classified by the challenges proposed to the practitioners and the taxonomy was constituted by the sport activities, group, modality and discipline. Surf is a discipline of surfing which is a sliding sport modality, therefore, a nature sport. In the context description, we had the wave’s components and constraints and the surfboards’ qualities. Through task analysis we obtained a taxonomy of surf manoeuvres. The structural and functional analysis allowed finding solutions for learning of surf techniques with trampoline and skateboards because these fit in sliding sports. MAST makes possible the development of strategies that benefit teaching and performance intervention.

## Introduction

Sports activities are in constant progression, allowing results to appear that initially seemed unthinkable. This progression is caused mainly by: the appearance of new techniques (ex. the Fosbury Flop in the high jump, appearing in the 1968 Olympic Games, as a new flight over the bar, introduced by Dick Fosbury, through which new records were achieved); rules modifications (ex. permission to play with feet in Volleyball, where originally it was played exclusively with the upper limbs); scientific development and technological progress, that had allowed to exceed demanding goals, coming close to the limit of the human body, which increases safety demands, and also greater support dependence (i.e. helmets, handles, boards, sails). During the sixties, there were nine meter waves ridden, which could not be exceeded until the appearance of the foot straps and the jet ski towin, that allowed to reach higher speeds and register a record of 23,77 m wave (Garrett McNamara in Nazaré, 2012).

In this context, qualitative research contributes in a positive way, insofar as it supplies events descriptions, in their natural settings, allowing for greater knowledge and a better professional preparation (Griffin and Templin, 1989). According to Thomas and Nelson (1996) and Culver et al. (2003) qualitative research is relatively recent in sport science although it has been sufficiently used in other areas such as anthropology, psychology and sociology. Qualitative analysis can be conducted based on observation, phase analysis, temporal analysis and critical features, but according to Lees’ (2002) investigations, information about the methods was not sufficient and, therefore, results could improve with an integration of methods.

Being this qualitative research, our focus was to develop a method to understand and explain sport activities, through its classification, with an ecological approach and having in consideration the characterization of environment, task and performers. The movements’ (techniques) systematization was accomplished with the structural and functional analysis of competitive practice. We aimed to construct a sport tasks matrix of analysis based on the taxonomic framing of the activities intending to improve the teaching and performance process. To proceed with an inductive process and to assess sports’ structure, at the beginning of our study we posed a major question: Is it possible to make a characterization of any discipline throughout the environment, tasks and performers analysis, in order to find solutions for an intervention in teaching and performance?

Surfing has developed significantly as a sport in the last few years, but there is a lack of applied research in human movement and performance analysis. Lowdon (1980) and Lowdon and Paterman (1980) were the first to study surfing in a sport science perspective researching physiological variables and the somatotype of international surfers. Recently this was improved by Mendez-Villanueva and Bishop (2005) with time motion analysis that led to new results in physiological aspects of surfboard riding performance but as they pointed out, further research was needed in all areas of surfing performance. According to Carr (1997) the surfer has to evaluate the wave and execute the manoeuvres in conformity, having in consideration that each wave has its unique characteristics compelling the surfer to a constant adaptation of different situations. The unpredictability of the environment implies a more in-depth study of it, aiming to transform the practice as predictable as possible, allowing to an evolution and also to the attainment of good results.

The physiological variables are performance indicators that aim to define some aspects of performance, but they should relate to a successful outcome (Hughes and Bartlett, 2002). For these authors the definition of useful performance indicators should be based on different structural definitions of games using match classification indicators, as well as technical and tactical indicators. To know and understand sport activities, it is necessary to describe the structure and relationships between objects to ease the manipulation of observations and as Fleishman and Quaintance (1984) state that is the purpose of classification. If these authors advocated that the taxonomy was more than mere classification, because it was the study of systematic classifications, including their procedures and rules, then the sport taxonomies could give deeper knowledge about sports structure.

In a top-down approach we should start with the sports classification because we need to understand the performance knowing what are the tasks and the environment categories including their constraints. Therefore, according to a bottom-up perspective we should begin with the task analysis to find out what is required to do (skills and motor patterns) and what are the performers needs to achieve tasks goals. In an ecological approach the constraining features of the task, the performer and the environment specify the choice that should be made by the performers (Davis and Burton, 1991). Considering the behaviour of sports performers as adaptive complex systems, Handford et al. (1997) argue that the appropriate solution to the task depends not only on task goals but also on the constraining features of the task, the performer and the environment. According to them in a constraints-led perspective of learning the practice design depends on appropriate manipulation of constraints related with sports characteristics. In this way practice is a continuous search for solutions with the choice that should be made by the performers based on available information and regarding the transfer of performance. If the performance outcomes are directly affected by the task or environment changing conditions, performers should know on advance what to expect. For that reason it is necessary to know which skills are available to overcome the challenges. Thus, we should consider the concept of ideal technique which is the best movement pattern based on scientific criteria, and the concept of goal technique being the optimal movement for each task as a result of dynamic interaction between ideal technique, the environmental situation and the capacities of the performer.

Task analysis can be defined as the process of identifying the components of a skill, movement or a cognitive process that a performer (or team) is required to follow in order to achieve a system goal (Davis and Burton, 1991; Kirwan and Ainsworth, 2001), furthermore, it is also a method that helps the researcher to collect information, organizing it and provides a detailed picture of that system which can be useful to skill and knowledge acquisition and performance assurance (Kirwan and Ainsworth, 2001). The analysis of sport tasks is a pertinent subject due to its importance in the teaching and learning process, that is, according to Chow and Knudson (2011) the better the tasks objectives are defined and also the factors that influence the technique, more easily is to intervene in the analysis and practice. This observation is in accordance with Lees (2002) that recalls researchers’ difficulties when intending to qualify the efficiency of the movement without appealing to the result. Thus, it is important to develop studies based on taxonomies and identification of the aspects that may imply motor actions, in order to be able to intervene in education and performance (Lees, 2002; Chow and Knudson, 2011).

The aims of this study were to improve sports knowledge with surfing characterization including all the manoeuvres and the environment constraining features and to find a way to describe the act of riding waves. The study was carried out creating surf techniques taxonomy through the Matrix of Analysis for Sport Tasks (MAST) which included sports taxonomy, tasks and environment description, structural and functional task analysis to finish with a proposal for teaching and performance strategies.

## Material and Methods

This was an interpretative study case with a qualitative method, studying the surf discipline in a competition flowing as the main activity. Data was gathered within the natural settings from 600 training sessions and 80 championships (National level, World Qualifying Series and World Championship Tour) which took place in Portugal. The subjects were 5 experts from the top 16 Portuguese surfers at that time training together with an average age of 23 years old and all of them having 9 years of competition surfing experience.

The study intended to use the Matrix of Analysis for Sports Tasks (MAST) and it was based on practice classification and task analysis. The MAST was applied in four phases: taxonomy; tasks and environment description; task analysis; teaching and performance strategies. Its application allowed the activities’ characterization through the observation, surfer’s opinions and bibliographical support. The triangulation of the data was used as an information data treatment.

The MAST construction was supported in six dimensions of analysis (purpose of analysis, subject matter, nomenclature, descriptive terms, structuring procedures, data sources and collection methods) each of them with many variations. This is useful to clarify the analysis method for different taxonomies of human performance (Patrick, 1992). The MAST aims to analyse the tasks, from a structural analysis where it is intended to analyse the process, related to the practice of a sport activity, in order to know it, as in the Vickers’ (1990) model, looking for an intervention to the level of education and performance. Considering that the analysis is task-oriented, a natural structure was defined, according to Sokal (1974) in Fleishman and Quaintance (1994), and supported by a steady and consensual terminology.

Framing the sport practice into a taxonomy, we accomplished data collection through observation and existing documentation, as to define the main characteristics and aims of the sports practice, compiling and interpreting the information found, being this a methodology defended by different authors (Denzin and Lincoln, 2000; Kirwan and Ainsworth, 2001).

In task and context description we explained the tasks using flow diagrams (Kirwan and Ainsworth, 2001). Surfers were observed during their practice and recorded with a camera (Sony CCD-TR913E, in a tripod). After the session the subjects were confronted with the registered material what resulted in the description of the tasks and environment, and then we complemented the gathered data with information collected throughout the bibliography. Observation and video analysis were carried out from a car using a portable DVD with an incorporated monitor of 7’(Scott DPX5), which was switched to a camera so recorded images could be reproduced. According to the aim of the training session we started by observing all ridden waves in real time followed by the observation of the most important performances in slow motion. The environment description was accomplished on the basis of the observation of Portuguese beaches during training sessions and each competition based on wave, wind and currents variables. The chosen boards by surfers to training sessions and competition were observed and related with practice conditions and performance.

Tasks analysis was accomplished based on the observation done during national and international competitions, using VHS images, a video recorder (Sony SLV-T2000) and a TV 37’ monitor (Philips CM8833). Intra and inter relationships were also examined through observation and feedback given by surfers, at the end of each training session, and complemented with bibliographic analysis. Knowing that there exists a sports category named sliding sports (practice with a linear displacement), after the analysis, we looked inside these sports modalities for some strategies to develop a training design, considering the task similarity and constraints manipulation, in order to simplify the task. We also studied other training methods in various activities, having in mind the aim and similarities of the movements.

To guarantee the internal validity we appealed for the interaction between the researcher and the group, and also the inferences between the researcher and observed subjects. On the other hand, for the external validity we used procedures’ documentation. The data collected from direct observation (long-term data collection and repeated observations), literature analysis, video analysis and inferences between researchers and observed subjects were confronted in order to reduce the bias, through data triangulation treatment, being this method recommended by several authors (Denzin and Lincoln, 2000; Griffin and Templin, 1989; Thomas and Nelson, 1996).

## Results

The presentation of the results is based on four phases of the MAST application: taxonomy, tasks and environment description, task analysis, and teaching and performance strategies.

### Taxonomy

Based on a classification of sports characteristics and performers practice we developed a taxonomy which was constituted by the following categories: sport activities, group, modality and discipline. The sport activities included individual sports, team sports, combat sports, racquet sports and nature sports. Within the nature sports, characterized by the practice against nature, three groups classified through practice characteristics were created: salience’s progression sports (e.g. climbing, trekking), free fall sports (e.g. paragliding, skydiving, BASE jump) and sliding sports characterized by linear displacement (Tomlinson, 1996; Brymer et al., 2009). In the last group, and depending on motor behaviour and context characteristics, we included the modality of skiing, snowboarding, wakeboarding, skating and surfing, characterized by the linear displacement in a wave, which comprised, depending on boards used and its types, the discipline of bodysurfing, body boarding, knee boarding, skimming, longboarding and surf characterized by the linear displacement in a wave with a short board.

### Environment and Task Description

**Figure 1:**
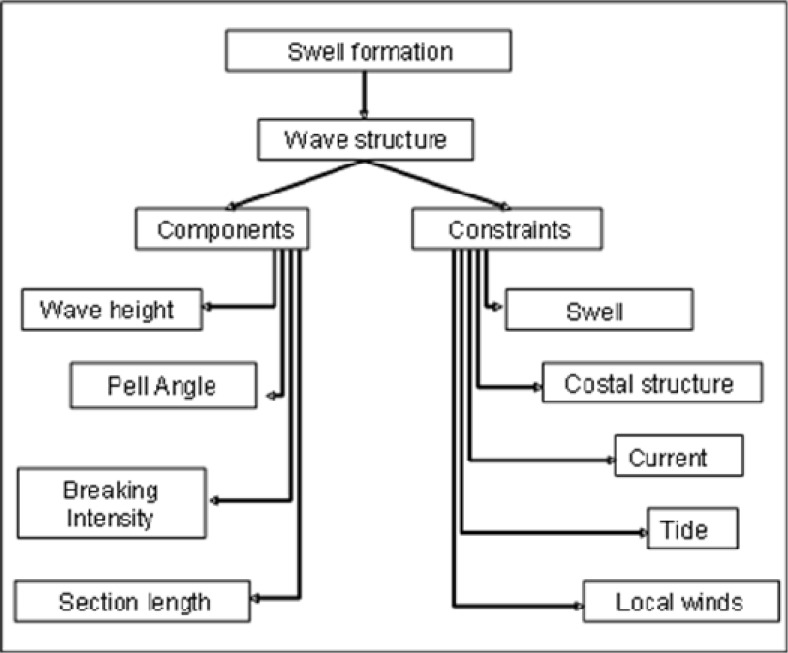
Wave components and constraints

In the environment description the waves and the surfboards were considered. The knowledge of the swell formation process allows the anticipation of the breaking waves (waves’ components and waves’ constraints) as can be seen in Figure 1. The waves are generated by the action of the wind blowing over the surface of the sea and propagate away as free-travelling swell (Butt and Russell, 2002). The practice of surfing is on a peeling wave, being therefore important to know the environment in the near shore zone where the wave begins to break: shoaling zone, breaker zone, surf zone and swash zone. Surfing waves start peeling from the breaking point along the wave crest (to the left or to the right) and the most powerful point is the pocket (Hutt et al., 2001). Characterization of surfing waves should be done through four wave components: height, peel angle, breaking intensity and section length as Mead and Black (2001a, 2001c) emphasized. We advocate that the characteristics of surfing waves change with the wave constraints that vary from location to location and day by day. With swell approaching the shore the wave’s quality changes within swell direction, coastal structure, currents, tides and local winds (Figure 1). The coastal structure includes coastal configuration, seabed types, seabed morphology and a slope of the beach (Mead and Black, 2001b). Knowing the wave’s behaviour, based on a characterization of surfing waves, surfers will have a quicker response on what manoeuvres should be performed.

**Figure 2:**
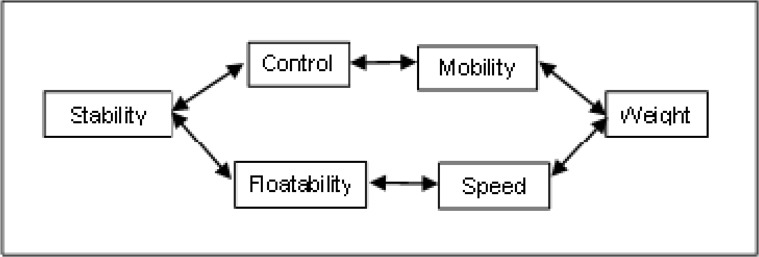
Surfboard qualities

As main surfboards’ characteristics we have the shape (size, template, section and rocker), the composition (material, cover) and the fins (number, placement, size, shape, composition and system), being all associated to the quality of the surfboard (Gudimetla et al., 2009). If one aims to learn a new manoeuvre or improve wave riding then some changes in the surfboards characteristics are necessary. With the surfboard characteristics we can find the qualities that we need for our surfboard, which should be different for each surfer. The capacity to maintain the surfboard in a specific position is called stability and it is related to control and floatability. Control is the capacity of driving the board and floatability is the quality that puts the board out of the water. The surfboard control is related with mobility which depends on board’s weight. The sliding speed not only depends on board’s weight but also on floatability (Figure 2). The surfboard size and template are related to stability. The section is related to floatability, mobility and speed while board’s foil is associated with control, floatability and speed. With changes in fins characteristics we can have a better stability, control or mobility and speed.

In surf we have a big number of techniques and most of them are manoeuvres (Mendez-Villanueva and Bishop, 2005; Everline, 2007). Before catching waves there are approach techniques including paddling to reach the take-off area and passing through waves (e.g. eskimo roll, seat down, diving, and most used duck diving) and waiting techniques (prone or seat down). For catching waves the most used technique is power paddling but it is also possible to perform the torpedo (fast change from seat down to prone position in wave’s face). The wave riding starts after catching the wave and through our tasks description 9 groups including several techniques were found (Table 1).

**Figure 3:**
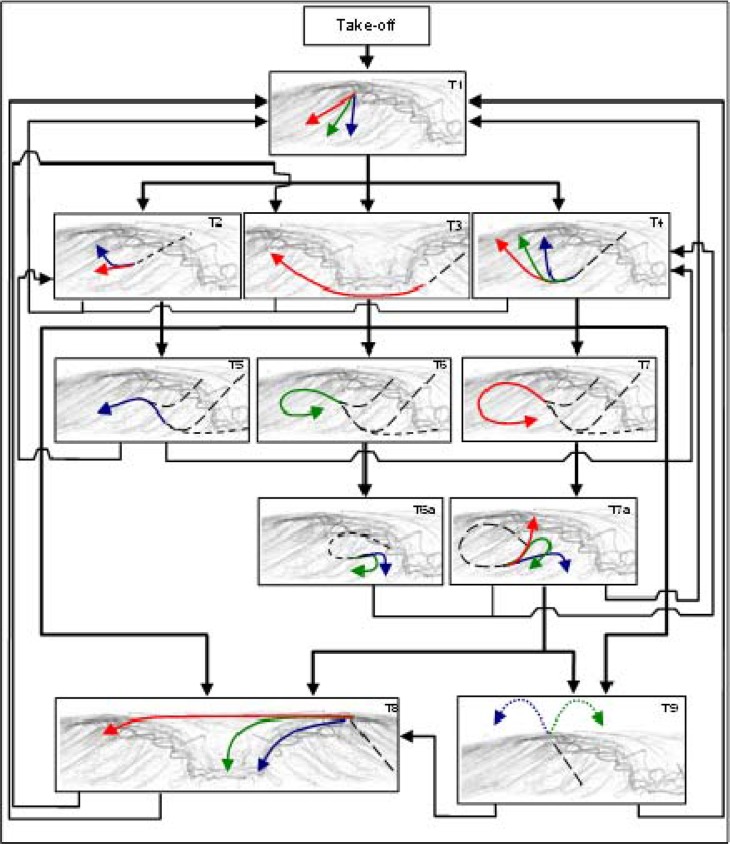
Trajectories in waves (Dash – done trajectory; arrows – possible trajectories) T1- Top down trajectories; T2- Mid face trajectories; T3- Down top passing section; T4- Down top trajectories; T5/T6- Mid face turns; T6a- Return trajectories; T7- Wave face turns; T7a- Rebound trajectories; T8- Sliding over trajectories; T9- Aerial trajectories.

The wave riding is a top down sliding with manoeuvres performed near the wave’s pocket until the end of the wave’s section, the wave breaks on the beach or if the surfer falls down. After the tasks description we were able to develop a flow diagram with the trajectories in waves and the possible linking between techniques (Figure 3). The wave riding starts with take-off going on with top down trajectories (T1) linked with mid face and wave face trajectories (T2) or bottom up trajectories (T3, T4), which begin with bottom turns. After the bottom up trajectories it is possible to do top turns, mid face and wall face turns (T5 to T7), sliding over the wave (T8) or out of the wave with an aerial situation (T9) and to end the riding anytime.

### Task Analysis

**Figure 4:**
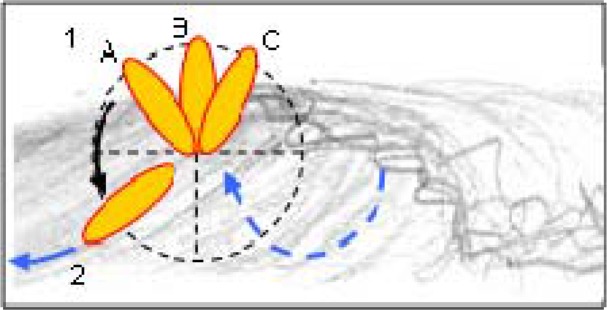
Surfboard movement (1A top turn, 1B vertical turn, 1C over vertical turn; 2 manoeuvre end)

A structural task analysis was carried out having in consideration several techniques related to the environment (wave and surfboard), searching for intra and inter task relations. A functional task analysis was done for better understanding of surfing techniques. The structural analysis allows better insights into top turns, sliding over the wave and airs. The surfboard’s angle on the wave’s top is important to differentiate the top turn manoeuvres (Figure 4). In the top turn the angle is less than 130º, in the vertical turn it is 180º and over 180º in the over vertical turn.

With different trajectories we can differentiate the manoeuvres from the group sliding over the wave (see M6 in table 1). All of them are floaters (lip floater; curtain floater; floater reentry) but different in goals and possibilities of linking with other manoeuvres because they are finished in different parts of the wave (see T8 in Figure 3).

**Figure 5:**
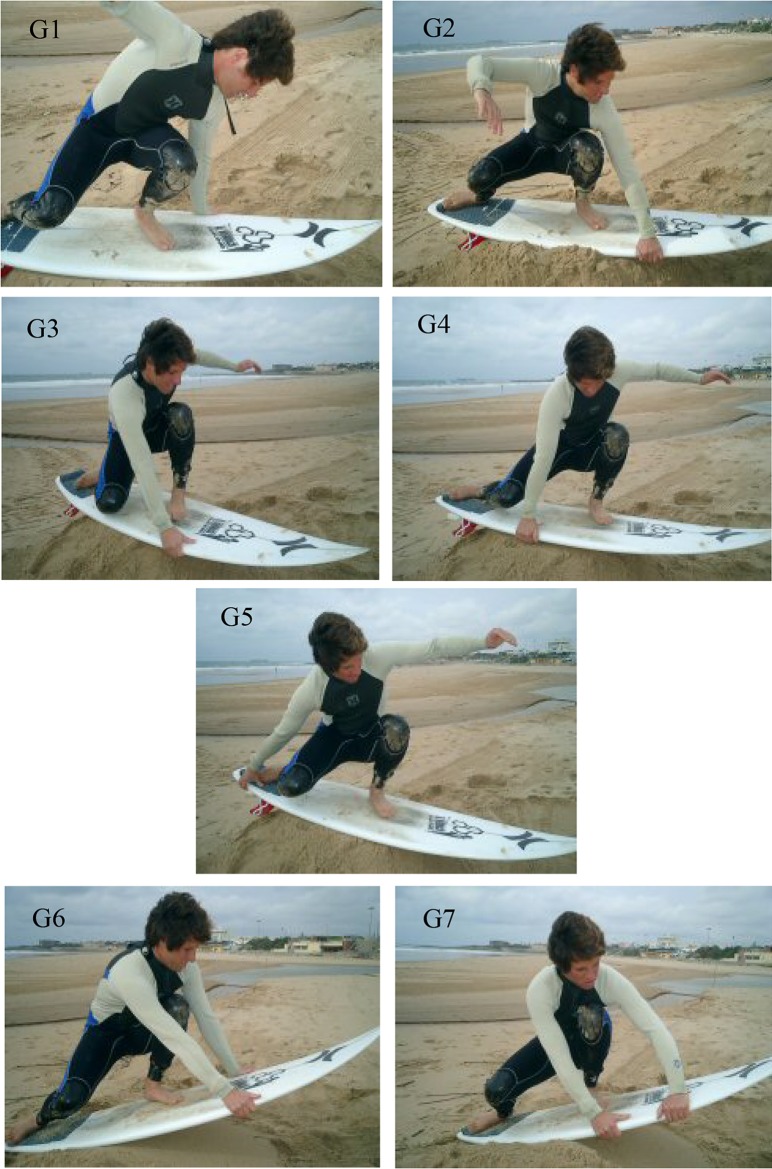
Types of surfboard grabs G1- Front foot forehand front arm one grab; G2- Front foot backhand front arm one grab; G3- Front foot backhand back arm one grab; G4- Between feet backhand back arm one grab; G5- Back foot backhand back arm one grab; G6- Mixed front double grab; G7- Front foot backhand double grab.

In the airs there are the natural and reverse movement together with the long axis and the transverse axis rotations. We also have the grabs which can differentiate the airs but should not be considered as new manoeuvres. The surfer can use (Figure 5) the back arm (G3 to G5) and the front arm with a forehand (G1) or a backhand grab (G2), near the nose (G3), in the middle (G4) or in the tale of the board (G5). It is also possible to use a double grab in both of the rails (G6) or in the same rail (G7).

Following Carr (1997) and Chow and Knudson (2011), we performed a functional analysis throughout the determination of the task aim and its characteristics, based on the context and techniques description, so that a standard executions study could be done, appealing to task division and to mechanical understanding of identified aspects. By dividing the techniques into phases it could be concluded that the final phase of one manoeuvre corresponds to the setting of the following manoeuvre.

Some characteristic aspects found through the functional analysis allowed better understanding of the techniques. In the take-off it is important to maintain the trunk parallel to the board to make possible the alignment between the surfer’s centre of gravity and the board centre of buoyancy aiming for speed. Also aiming for the sliding position and sliding over the waves, the alignment of the surfer’s centre of gravity with the front foot over the board centre of buoyancy is fundamental. In order to perform a good turn, sliding speed is necessary, therefore, the surfer’s position with the trunk laying down to the inside of the curve, creating an optimal centre of gravity positioning away from the board, is also very important. The magnitude of centrifugal force is dependent on the shape and the speed of the turn. Thus, the centrifugal force will be higher with faster sliding and a sharper turn and then the angle of the resultant force vector will be larger (Carr, 1997; Muller et al., 1998). If the speed is higher the surfboard should have a bigger angle and needs to be with the inside rail in contact with the wave. This is the reason why carving (a sharp inclination turn, executed with great speed and one of the edges of the board submerged) is not a manoeuvre but a degree of difficulty of turning being synonymous with a fast and sharp turn.

In airs it is very important to control the centre of gravity trajectory and the reception on the wave searching for the alignment between the surfer’s centre of gravity and the board centre of buoyancy aiming for balance control (dynamic equilibrium).

### Teaching and Performance Strategy

The relationship between modalities of the same group (sliding sports) or disciplines of the same surfing modality, allows to find similar tasks providing transfer situations and facilitating the learning process namely with the use of skate or Malibu boards. According to Abernethy et al. (2005) this transfer makes it easier to introduce new tasks and speed up the learning process. Having as a reference mechanical understanding argued by Carr (1997) and Tchórzewski et al. (2013), as well as a facilitated learning process based on transfer situations, we must reduce stability (to improve dynamic equilibrium) using a smaller base and a greater height of the location where the take-off happens, introducing later on the slide in the terrestrial environment, so that we finish by executing the task in the aquatic environment (first in the foam followed by the wave’s face).

**Figure 6:**
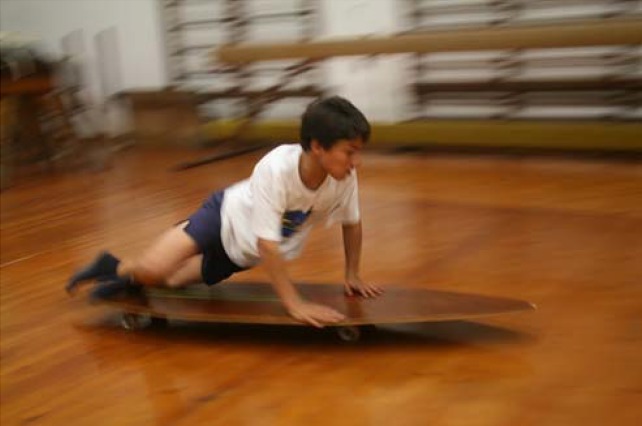
Take-off training with slide in the terrestrial environment using a take-off skate

As main teaching and performance strategies for take-off learning, the board bag on land is used to understand the movement and the bench take-off to introduce more instability. The next step should consist of introducing a sliding situation with the take-off skate (Figure 6) and only afterwards a sliding situation in the wave’s foam. Braking wave quality also should be considered as a way of progression, as well as the boards with different characteristics.

**Figure 7:**
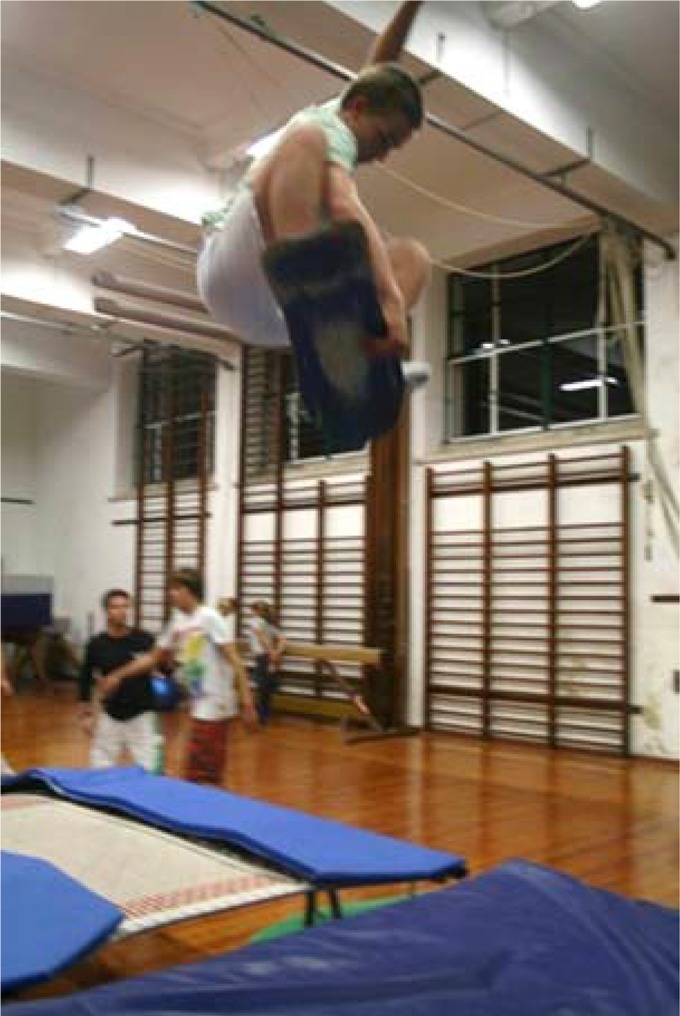
Air training with a skateboard in a double mini trampoline

For bottom turns and top turns fitness instable boards and special skates prepared to carving turns are used. If the aim of a good air is controlling the centre of gravity trajectory then the best teaching and performance strategy relies on trampoline or double mini trampoline training sessions. This kind of gymnastics apparatus are also good for training the reception on the wave aiming for balance control and the alignment between the surfer’s centre of gravity and the board (Figure 7).

## Discussion

According to Pinder et al. (2011) researchers must evaluate and characterize the competitive environment so that this can be replicated in training. Through the construction and application of the MAST we obtain a qualitative method to initiate some studies on sports to serve as a base for new investigations and new training designs. With this specific classification we can characterize a sport activity in its natural setting and find similarities between different modalities that allow setting correspondences (training and competition). Beside this characterization we also performed the task analysis which allowed the study of task performance as suggested by Araújo et al. (2007)

The elements are classified by the challenges proposed to the practitioners and the taxonomy is constituted by the sport activities, group, modality and discipline. The taxonomy allows to understand that surf is a discipline of surfing which is a sliding sports modality, therefore, a nature sport. These results corroborate with some other authors’ perspectives about practice intervention (Lees, 2002; Chow and Knudson 2011). According to Lobato (2006) the transfer from the actor-oriented perspective is verified in new situations depending on previous experiences which in practice can be enhanced by observed similarities and also by the practice of different sports.

By characterizing surfing waves, we were able to formulate some guidelines in order to predict wave’s behaviour which not only allows a better choice of training locations but also gives surfers an idea on what manoeuvres to perform. Furthermore, with surfboards characterization it was possible to understand and combine different surfboards’ variables in order to develop a learning process. This proposal of technical progression is at the same time a method to improve high level surfing. The waves and surfboards characterizations allowed the identification of environment variables leading to managing the learning and practice environments for a replication of performance environments as proposed by Renshaw et al. (2010). The techniques’ description made possible creation of a classification and hierarchical relation between surfing manoeuvres which allowed a better design of practice tasks and better surfer’s determination of affordances to overcome the wave challenges. These are key points to ensure total understanding of surfing constraints and how the performance environment in training sessions for surfer’s perception-action coupling could be replicated as mentioned by Pinder et al. (2011).

The structural analysis allowed to identify and explain the inter task relationships (connections between different groups of manoeuvres) as well as intra task relationships (connections between manoeuvres of the same group). The functional analysis not only allowed better understanding of the techniques, but also helped to justify that carving, layback, off the lip and grabs, instead of manoeuvres set degrees of difficulty. With task analysis it is possible to observe the relationship between: manoeuvres and the sections of the wave; surfboards and the waves; manoeuvres and the technical level. The structural and functional analysis allows task and environment simplification which ensures skills development in the real-world as mentioned by Brymer and Renshaw (2010), but also makes possible the task decomposition that could be important to dry land training sessions. To implement a functional variability in movements in order to achieve consistent outcomes in different, often unpredictable, outdoor contexts as suggested by Brymer and Renshaw (2011), understanding and learning of the manoeuvres must be from the terrestrial environment to the aquatic one, from a stable context to an unstable one, from a plain surface to an inclined one. The solutions found for learning and training of surf techniques (i.e. airs using gymnastic skills on trampoline) were achieved with the structural and functional task analysis. In the same way it is possible to use skateboards because they fit in sliding sports which can be a guarantee of transfer of knowledge between tasks and allows learning and repeating performance outcomes consistently although a movement pattern has some changes. These proposals and learning design solutions with “controlled boundaries of exploration in dynamic settings” would facilitate new movement solutions as suggested by Brymer and Renshaw (2010) in the constraints-led approach to learning in outdoor education.

In conclusion, sport taxonomies can provide deeper knowledge of sports structure and the MAST (helps to find environmental, performer and task constraints) makes possible the development of strategies that benefit teaching and performance intervention. To accomplish the tasks in surfing it is necessary to perceive the action process and the study of surf riding could improve this knowledge. Using this approach, we found a teaching strategy based on manoeuvres progression that helps to construct a Long Term Surfers Development.

In further research this study might be the basis for investigations aiming to characterize a sport and where predictors of performance are searched for. Further research is needed taking into consideration a quantitative approach in order to analyse similarities between tasks and required skills as well as similarities between artificial (laboratory and training sessions) and natural settings (competition).

## Figures and Tables

**Table 1:** Manoeuvres Groups

M1	M2	M3	M4	M5	M6	M7	M8	M9
Take-off	Sliding on the Wall	Bottom turn	Middle turn	Top turn	Sliding over the wave	Airs	Sliding inside the wave	Final
Take-off	Drop	Bottom Turn	Fade	Top Turn	Curtain Floater	Air	Cover Up	Kick out

Angled Take-off	Angled Drop	180º	Pump Turn	Vertical Turn	Floater Reentry	90º	Tube	Pull out

Vertical Take-off	Reverse Drop	Reverse 180º	Mid Face Turn	Over Vertical Turn	Lip Floater	Reverse 90º	One Grab	Step off

	180º Drop		Cut Back	Extended Vertical Turn	Foam Floater	180º	Stand Up	Nose dive
			
	Reverse 180º Drop		Round house	Snap	Reverse 180º	Reverse 180º		Bail Out Dive
					
	Air Drop		Rebound	360º	360 out	Reverse 270º		Bail Out Jump
					
	Trim			Reverse 270º	Lip Slide	360º		Uncontroll Wipe Out
						
	Hopping			Reverse 360º		Reverse 360º		
								
	Stalling					90º Switch Stance		
								
						Reverse 180º Switch Stance		
								
						Long Axis 360º		
								
						Transverse		
								
						Axis 90º		
								
						Transverse Axis 360º		
								
						Transv90º Frontal90º		
